# Enhancing maternal health service utilization among highly vulnerable pregnant women through a human-centered design process: Study protocol for a quasi-experimental study in Oromia, Ethiopia

**DOI:** 10.12688/gatesopenres.16277.1

**Published:** 2024-08-27

**Authors:** Bee-Ah Kang, Habtamu Tamene, Yihunie Lakew, Daryl Stephens, Rajiv Rimal

**Affiliations:** 1 Department of Health, Behavior and Society, Johns Hopkins University Bloomberg School of Public Health, 624 N. Broadway, Baltimore, MD, 21215, USA; 2 Center for Communication Programs, Johns Hopkins Bloomberg School of Public Health, Africa Avenue (Bole Road), Dembel City Center 10th Floor, P.O Box: 26171, Addis Ababa, 1000, Ethiopia

**Keywords:** maternal and child health, vulnerability, human-centered design, research protocol

## Abstract

**Background:**

Disproportionate uptake of and access to maternal and child health services remain significant challenges across and within countries. Differing geographic, economic, environmental, and social factors contribute to varying degrees of vulnerabilities among individuals, which manifest as disparities in maternal and newborn health outcomes. Designing solutions according to need is vital to improve maternal and child health outcomes. In this paper, we describe our study protocol on evaluating the effectiveness of an intervention package developed through a human-centered design (HCD) process in improving maternal health service uptake among vulnerable pregnant women in rural areas of Ethiopia.

**Methods:**

The study has two distinct phases. For both phases, solutions will be developed leveraging HCD approaches to improve institutional delivery and antenatal care uptake among vulnerable pregnant women. Final solutions will be implemented in collaboration with community health workers and health officers. A community-based, quasi-experimental, mixed-method study design is used to assess differences between intervention and control arms. A panel sample will be enrolled after screening for pregnancy and vulnerability level and surveyed at baseline and midline in Phase 1. In Phase 2, a post-only study will be conducted in the same intervention and control sites. Pregnant women will receive refined solutions for six months, which will be evaluated using end-line assessment. Qualitative interviews will be conducted among vulnerable pregnant women and healthcare providers.

**Conclusions and Implications:**

Our sequential approach to evaluating initial solutions, which in turn will be used to inform the enhancement of solutions, will provide practical insights into how solutions are accepted among vulnerable women and how they can be better integrated into women’s lives and health systems. Our findings on program effectiveness and implementation research will be disseminated to the Ethiopian Ministry of Health and its partners to inform large-scale implementation at the national level.

## Introduction

Reducing maternal mortality ratio (MMR) has been one of the major priorities on the global health agenda. With the launch of the Sustainable Development Goals (SDG) in 2015, the World Health Organization (WHO) and partners reached a consensus on endorsing Target 3.1 that aims to reduce the global MMR to less than 70 per 100,000 livebirths by 2030. The target has shown a steady progress with its 34.3% reduction between 2000 and 2020 ^1^. However, such aggregated outcomes in health indicators often obscure disparities across countries and within sub-regions.

Ethiopia has shown steady improvements in many maternal and child health indicators over the past two decades. In 2000, the MMR was 871 deaths per 100,000 livebirths, and declined to 412 in 2016 ^2^. Between 2005 and 2019, under-5 mortality also decreased by 52% ^3^. Despite this substantial progress, Ethiopia faces persistent health challenges. The MMR in-country accounts for 3.6% of global maternal mortalities ^4^. Twenty-five percent of female deaths from the 2016 data were found to be from pregnancy-related causes ^2^, and the neonatal mortality rate also increased by four points per 1,000 livebirths between 2016 and 2019 ^3^.

To achieve faster and more equitable improvements in maternal and child health outcomes, the government of Ethiopia has implemented a three-tiered health system with an intensive Health Extension Program, which takes a community-based approach to improving health knowledge, health-related skills, and access to primary healthcare ^5^. In addition, the Health System Transformation Plan (HSTP-II) is being implemented from 2020 to 2025 with an overall objective to improve the population’s health status by accelerating progress towards universal health coverage, protecting populations during health emergencies, transforming woredas toward zero home delivery, and improving health system responsiveness ^6^. The HSTP-II has a target to reduce the MMR to 279 per 100,000 live births and under-5 and neonatal mortalities to 44 and 21 per 1,000 live births, respectively ^6^. Under these national goals, providing pregnant women with essential maternal health services, including institutional delivery (i.e., childbirth occurred at health facilities with skilled birth attendants), antenatal care (ANC), and iron and folic acid (IFA) supplementation, has been a key strategy for optimizing maternal and child health outcomes in Ethiopia. These maternal health services can significantly reduce the risk of maternal, perinatal and neonatal mortality in low- and middle-income countries (LMICs) and alleviate health disparities worldwide ^7– 9^.

Nevertheless, there remains an urgent need to address disproportionate uptake of and access to maternal health services in Ethiopia. Taking institutional delivery coverage as an example, women’s use of health facilities for childbirth greatly varies by region and sociodemographic characteristics. With the national average being approximately 48%, the percentage of institutional delivery use ranges from 23.3% in Somali Region to 94.8% in Addis Ababa, the capital city ^3^. Also, 79% of women in the lowest wealth quintile delivered a baby at home; the corresponding figure for women in the highest wealth quintile was 14%. Parity is also associated with home delivery: women having six or more births are more likely to deliver at home, compared to those delivering for the first time.

Prior evidence suggests that women’s use of maternal health services is shaped by multiple factors at the socio-ecological continuum. Barriers to maternal health service uptake among pregnant women reside at multiple levels, including at the individual level (having poor knowledge of obstetric complications and danger signs, low risk perceptions, high parity, and no birth preparedness plans) ^10– 12^, social level (social and gender norms, support from family, decision-making power) ^11, 13, 14^, cultural level (respect for elders, perception about pregnancy-related matters as women’s privacy, relationship with traditional birth attendants in the community) ^3, 15– 17^, structural level (distance to health facilities, transportation and road infrastructure, costs for traveling) ^11, 15, 18^, and those that are service-related (lack of services that respect women’s privacy and preferences, disrespectful treatment by health workers, and staff and medical supply shortages) ^10, 15, 19, 20^.

Understanding vulnerabilities of key target populations has important implications for developing and implementing tailored programs and policies ^21^. Nevertheless, less attention has been paid to how vulnerability is defined, conceptualized and operationalized in the realm of maternal health service coverage ^22^. Indeed, despite remarkable improvements in disease-focused service coverage, global health programs have been highlighted as neglecting inequity by not reaching the most disadvantaged population, straining weak local health systems, and distorting local health priorities and agendas ^23, 24^. Our project is based on the assumption that accurate and comprehensive assessment of vulnerability in maternal service uptake can help identify vulnerable pregnant women with the lowest likelihood of accessing necessary health services, which will subsequently help generate evidence and provide guidance on effective policies and interventions. Therefore, identifying the most vulnerable pregnant women and engaging them in program development would enhance the applicability, viability, and effectiveness of an intervention. Adopting user-oriented strategies, our study attempts to render vulnerable pregnant women an opportunity to take ownership of their problems and participate in generating solutions with the study team to cater to their unique needs, challenges, and hopes.

Furthermore, we argue for the need for conducting implementation research, given that determining *true* program effectiveness necessitates evidence on how an intervention fits in “real world” settings beyond a controlled study environment. Implementation research is distinguished from monitoring or process evaluation efforts due to its focused goals to explore 1) a program’s likelihood of being adopted and scaled up in real-world settings, 2) contextual factors that may facilitate or hinder implementation, 3) implementation strategies (rather than intervention strategies), 4) and the engagement of stakeholders to achieve successful implementation ^25^. Well-designed implementation research provides an insight into whether an intervention would likely be integrated into its target environment, and more broadly, into health systems beyond study duration and scope. Implementation research also generates evidence that helps policy makers and implementers foresee challenges, contributing to saving costs and resources for future programs. Although there is no scientific consensus on what elements and principles are necessary for conducting implementation research, the WHO guide ^26^ encourages researchers to outline implementation research questions, use a framework that describes implementation research outcomes, and select a study design and methods that are most pertinent to understand how implementation occurs in a given context.

### Study objectives

The objective of this study is to evaluate the effectiveness of an intervention package, developed through a human-centered design (HCD) process, in improving maternal health service (institutional delivery and ANC) uptake among highly vulnerable pregnant women living in rural areas of Ethiopia. Specifically, the study objectives are:

1. To assess whether changes in women from baseline to end-line in the intervention arm are significantly greater than corresponding changes in the control arm in the following outcomes: (a) institutional delivery and (b) ANC visits.
2. To explore how social and behavioral factors, including social norms, gender norms, women’s empowerment, and spousal dynamics, are associated with maternal health service uptake.
3. To understand the extent to which the intervention meets key implementation research outcomes, including fidelity, acceptability, feasibility, and sustainability, among pregnant women and health workers in the intervention arm.

## Methods

### Study setting

Our study sites are in Oromia, the most populous region in Ethiopia. Within this region, we selected two woredas (administrative units below the zone, which is below the region) for our intervention sites. Sirraro and Shalla woredas were selected as intervention arms, and Gera and Shebe woredas as control arms. The study sites were not randomly selected due to the on-going security issues in Ethiopia.

### Study design

A community-based quasi-experimental study design was chosen to assess differences in key variables between the intervention and control arms. We chose a quasi-experimental design for several reasons. We lacked information about clear randomization unit given the characteristics of individuals or clusters (woredas), and it was likely that many woredas in Oromia region are not homogenous towards our outcomes of interest. Another reason was due to resource constraints, as we did not have a sufficient number of woredas in our study to conduct a randomized trial design. The study included two waves of data collection from a panel of recruited pregnant women at Phase 1. We attempt to leverage Phase 1 findings to inform the development of study tools and identify factors affecting implementation to provide insights into the improvement our intervention at Phase 2. This study is registered at ClinicalTrial.gov (NCT05907720).

We conducted the baseline survey of pregnant women enrolled in intervention and control arms in January 2024 and are in the process of conducting a midline assessment for Phase 1. After the baseline survey, we implemented a set of HCD prototype solutions in the intervention arm only, keeping standard of usual care intact in the control arm. After the 4-month period of HCD solution implementation, we are conducting the midline survey among the same women in the panel from both the intervention and control arms. The midline assessment includes in-depth interviews with purposively selected pregnant women from the panel and health workers who engaged in implementation.

**Figure 1.**
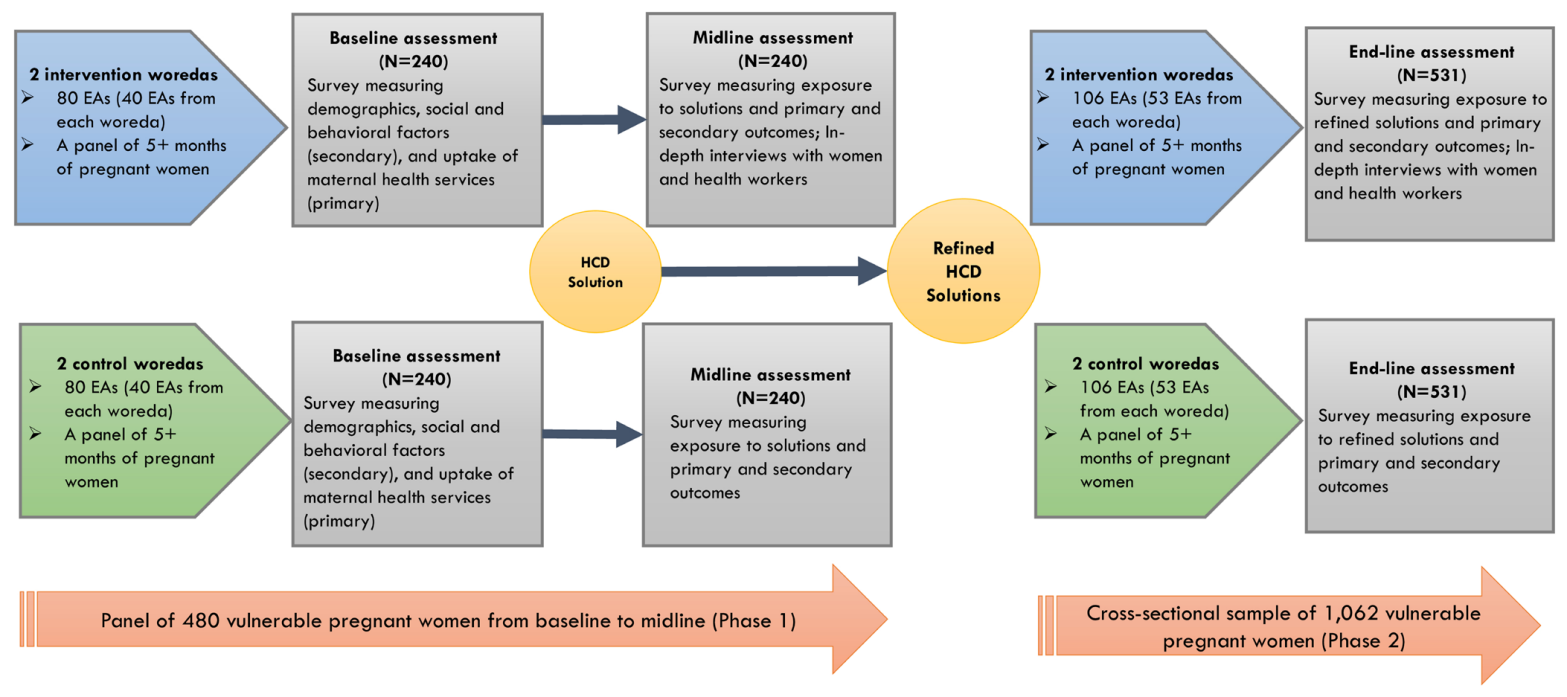
Quasi-experimental evaluation study among vulnerable pregnant women.

In Phase 2, we will conduct a post-only study with a new sample of vulnerable pregnant women who will be recruited after the implementation of refined HCD solutions. We will apply the same recruitement procedures as those for Phase 1. We will compare outcomes between the intervention and control arms at end-line, and the end-line findings will also be compared with baseline to identify whether refined solutions bring greater impact than initial solutions did in Phase 1. Thus, our overall study design includes a total of three assessments, baseline and midline assessments in Phase 1 and end-line assessment in Phase 2. The study design is further described in Figure 1.

### Sample

Our primary target participants are vulnerable pregnant women aged 15–49. For consent administration, we segmented adult pregnant women aged 19 and above, pregnant adolescent girls aged 15–18, and service providers. Service providers, such as midwives, Health Extension Workers (HEWs) and Health Development Armies (HDAs) who engage in the study will be recruited for qualitative assessment at midline (Phase 1) and end-line (Phase 2). Table 1 below summarizes inclusion and exclusion criteria for each sample population.

**Table 1.** Summary of inclusion and exclusion criteria of study sample.

Respondent type	Inclusion criteria	Exclusion criteria
Pregnant women	Women in their 5th or higher months of pregnancy; meet the high vulnerability criteria.	Pregnant women who are less than 5 months pregnant; meet the low vulnerability criteria.
Women who delivered recently (Midline and end-line only)	Recently delivered women included in the panel for baseline assessments during Phase 1; those who have received HCD solutions during Phase 2; meet the high vulnerability criteria.	Recently delivered women who were not included in the panel; did not have exposure to HCD solutions; meet the low vulnerability criteria.
Heath Extension Workers	Who work in health promotion and essential maternal health services in heath posts.	Who have worked in this position less than 6 months in the intervention arm.
Health Development Armies	Community volunteers who work on early screening and health promotion activities for pregnant women.	Who have worked in this position less than 6 months in the intervention arm.
Head of woreda health office	Head/Vice Head of the woreda Health Office who help facilitate implementation of the project	Who have worked in this position less than 6 months in the intervention arm.

### Sample size calculation

We considered institutional delivery (a major outcome variable) as a basis for the sample size calculation. To estimate sample size, we took account of our longitudinal design that measures the same subjects before and after the intervention and assumed an intervention effect of 9% in improving institutional delivery beyond the current 48% ^3^. Assuming a p-value of 0.05, interclass correlation coefficient (ICC) of 0.009, a power of 80%, a total of 240 pregnant women from the intervention arm and another 240 pregnant women from the control arm are needed, for a total sample size of 480 pregnant women in each study phase. The assumptions of sample size also took account of feasibility and sample characteristics. The number of enumeration areas (EAs) this phase can reach is 80 per study arm, and approximately 5 women are expected to be eligible in each EA. Based on the cross-sectional study design in Phase 2, we assumed 9% detectable difference between the study arms with an 80% power, which led to a sample of 531 for each study arm, taking account of 10% non-response rate. Approximately 106 EAs will be reached from given the number of eligible women in each EA. Both intervention and control arms will have an equal sample size in each phase, having 1:1 ratio of women between the intervention and control arms. As such, the total number of women to recruit across study phases is 1542.

For qualitative assessments, a total of 32 health workers engaged in implementation will be purposively sampled for in-depth interviews at post-intervention assessment in each phase. The interviews with health workers will explore the feasibility, acceptability, and scalability of the prototype solutions along with perceived program effects. An equal number of health workers will be interviewed at midline (post-intervention of Phase 1) and end-line (post-intervention of Phase 2). The interviewees selected for Phase 2 may be the same service providers recruited from Phase 1 depending on their availability and engagement in the program. We have chosen 32 as the target sample size based on our best estimate about achieving data saturation. In each phase, we will also interview 20 pregnant women from the panel who recently delivered and were exposed to our HCD solutions. The qualitative iterviews will be conducted only in the intervention sites.

### Recruitment

After obtaining a list of all interested women from HEWs and HDAs, data collectors will visit the women in person to screen their pregnancy status and vulnerability level to assess their eligibility. With the woman’s verbal consent, the data collector will administer the pregnancy screening tool to identify her pregnancy status. Only women 5+ months of pregnancy and aged 15–49 are considered eligible. If eligible, she will then be screened for her vulnerability level, assessed through the vulnerability screening tool. Those considered to be moderately to highly vulnerable are eligible and entered into the sampling frame. All responses to the screening questions will be entered into the data collectors’ mobile phone application that automatically determines pregnant women’s eligibility during the visit. Women who score at most 13 out of the maximum 20 vulnerability screening questions will be identified for the study participant. For the qualitative assessment, recently delivered women who receive our intervention and health workers in the intervention sites will be recruited. Purposive selection of the sample will take into account the extent to which women are exposed to the intervention. We will prioritize recruiting women in earlier gestational ages (5 ^th^ month or earlier) since women in late pregnancy terms may not be fully exposed to our solutions.

All eligible women will be entered into our sampling frame. After listing eligible pregnant women, we aim to recruit approximately three women per EA to achieve our target sample size. If an EA has five or fewer eligible women, they will be all contacted for recruitment. If there are more pregnant women than the minimum required number of pregnant women, we will randomly select the required number. If a woman is not interested or available for participation, we will contact the next randomly selected woman for recruitment. Recruitment will continue until our sample size is reached, expanding to other EAs if necessary. Recruitment in Phase 1 has been completed in this way, and Phase 2 recruitment will follow this procedure.

### Data collection and measurement

***Survey interview*.** All participant from Phase 1 and 2 will undergo a one-on-one survey interview to assess sociodemographic information, psychological factors, social and structural factors, and maternal health service utilization. A structured interview will be administered by a local data collector in the local language. The survey will capture the self-reported place of the most recent childbirth along with uptake and frequency of ANC visits during the most recent pregnancy. Secondary outcomes include knowledge, attitudes, and perceptions about maternal health service utilization, social norms, gender norms, spousal dynamics, decision-making, and community and structural factors. Midline (post-intervention in Phase 1) and end-line (post-intervention in Phase 2) assessments will additionally include questions measuring program exposure.

***Qualitative interview*.** Purposively selected women from the study participants (n=20) along with health workers (n=32) engaged in the program will participate in an in-depth interview. Given the 4-month duration of program implementation among pregnant women in their 5 ^th^ or higher months of pregnancy, interviews will be administered to recently delivered women inquiring about their perceived effects of the intervention on the place of childbirth and ANC uptake during pregnancy. Additionally, the interview guides for women and health workers will contain question around key implementation research outcomes, including acceptability and feasibility, to identify any similarities and differences arising across different types of interview participants.

### Intervention

***HCD workshop procedure*.** In Phase 1, prior to the HCD process, an extensive desk review and secondary data analysis were conducted to identify key drivers of vulnerability affecting uptakes of maternal and child health services. Vulnerability driving factors, such as women's illiteracy, distance from health facilities, high parity, decision making power, exposure to media, and lack of household assets were identified as major barriers to maternal health service use. Based on these factors, a vulnerability screening algorithm was developed to identify the target population of rural vulnerable pregnant women. From the evidence synthesis, the two initial design challenges focusing on ANC and institutional delivery were also prioritized: 1) reimagining how to support pregnant women to attend ANC, and 2) reimagining how to support pregnant women to deliver in a health center. Then, a five-stage design thinking process was implemented, using empathy, definition, ideation, prototyping, and testing stages. In this process, we engaged key stakeholders, such as vulnerable pregnant women, their husbands, and healthcare providers in co-creation workshops.

The co-creation workshops were separately organized for vulnerable pregnant women, husbands of vulnerable pregnant women, and healthcare providers to form homogeneous groups and avoid power dynamics for better flow of information and ideas. A total of eight design teams, each comprising six to eight design team members, went through the design thinking process for three days. A core team that involved senior design staff further synthesized the outputs of the workshops. Insights around limited household support for pregnant women, low awareness on importance of healthcare services, demotivated healthcare providers, and poor service quality were harvested from these workshops.

***Development of HCD solutions*.** In the prototyping phase, our team prioritized potential prototype solutions based on an impact and feasibility matrix. The matrix served as a grading system to narrow down prototype solutions from those that were initially ideated among workshop participants based on the two dimensions. According to participants’ perceived impact (low or high) of solutions on institutional and ANC uptake, along with perceived feasibility level (less or more likely to be implemented) pertaining to resource constraints, three solutions were selected: audio programs that encourage couple communication, visual print materials, and home-to-home visits by community health workers.

The study team developed two pre-recorded audio programs containing real stories of pregnant women in the local language of the target area. The first audio session focuses on the importance of ANC through the narration of a couple's story, highlighting their communication and the husband's support in household tasks, as well as accompanying the wife to ANC visits. The second audio session focuses on the importance of institutional delivery from a story of a pregnant woman. It describes her first childbirth at home, which was a painful and difficult experience. The story then progresses to her second childbirth experience at a health facility, where she was well-prepared and supported by her husband. Furthermore, the solutions included two visual print materials (pamphlets) with contents that reinforced the audio messages about ANC and institutional delivery. These prototypes were tested through focus group discussions with pregnant women, their husbands, and community volunteers, and then refined based on user feedback. All five of the design thinking stages were implemented in the two study sites (Shalla and Siraro) of Oromia regional state.

For Phase 2, we aim to leverage implementation research data obtained from midline qualitative interviews at Phase 1 to finalize our HCD plans. Solutions from Phase 1 will be revisited by a newly organized design team to collectively explore the most effective and feasible implementation strategies. We will conduct the five-stage HCD process, sharing Phase 1 findings with workshop participants and engaging prior end-users. Since Phase 2 will focus on refining and strengthening the existing solutions, some of the HCD activities (e.g., “How Might We” statements and ideation) are expected to center around establishing strategies for improving the materials, delivery, and format of the solutions, ultimately to integrate our solutions into the existing health system.

### Implementation plan

As part of the implementation of the interventions in Phase 1, the study team conducted an orientation training for HEWs and HDAs (known as Hadha Gare locally) to equip them with the skills needed to identify vulnerable pregnant women using a vulnerability screening tool and to effectively implement the solutions. The prototype solutions were delivered by the trained HDAs with the help of HEWs for a duration of four months from February to May 2024.

Prior to implementation, HDAs identified vulnerable pregnant women within their catchment area. They then scheduled an appointment to facilitate family discussions about the prototype solutions. The audio programs and print materials were intended to be delivered throughout five home visits by HDAs. For women whose gestational age was high (8-9 months), we aimed to provide audio and print materials on institutional delivery only through three home visits.

In the first visit, HDAs provided the household with an audio device containing the pre-recorded ANC program and gave instructions on how to engage in couple communication. After two to three days, HDAs revisited the household to inquire about questions or concerns from the couple, collected the device, and provided a print material to reinforce the audio content. HDAs then conducted a third visit to gather feedback from the couple and provided the second audio device that contains a story of institutional delivery. At the fourth visit, HDAs collected the second device and provided the second print material to reinforce institutional delivery messages from the audio program. Finally, HDAs conducted the follow-up visit, encouraging the pregnant woman to utilize health services until she delivers and addressing remaining concerns among the couple. All of the home visits required husbands’ presence given that the solutions were designed to facilitate couple communication, which would in turn optimize male involvement in maternal health service potentially through shifting social and gender norms.

To ensure seamless implementation of the interventions, the study team conducted close monitoring of activities that included site review meetings with implementing partners at the primary health care unit (PHCU) level and on-site supportive supervision to identify gaps, provide technical support, and address any identified implementation challenges. Key implementation outcome indicators, such as the number of pregnant women reached with ANC and institutional delivery audio programs and print materials, were regularly collected through the KoboCollect mobile platform.

The Phase 2 implementation plan may follow a similar procedure. Detailed plans will be determined based on information about how the current implementation scheme was acceptable and feasible among health workers and implementing partners. Specifically, the Phase 1 qualitative interview data on implementation research indicators will inform Phase 2 strategies.

### Data analysis plan

***Quantitative data analysis*.** First, we will conduct a series of bivariate tests, including chi-square and t-tests, across treatment and control arms based on baseline and endline data. The purpose of conducting bivariate tests is to identify differences between the study arms for each potential confounding factors, given the possibility of quasi-experimental design not ensuring baseline matching between intervention and control arms. If any differences are observed, they will be controlled for in subsequent regression analyses. We will also identify types of missing data to determine whether missingness occurs in a systematic manner, which will help us determine the appropriate remedial measure to adopt.

Since this project will conduct various implementation activities at the PHCU level, the analysis will take account of clustering at the level. Further, because *kebeles* (akin to villages) are clustered within PHCUs, we will also consider clustering at the *kebele* level. Given the nested nature of this study, multi-level modeling (MLM) is considered appropriate because it provides both cluster-specific and population-averaged estimates simultaneously by defining both random effects and fixed effects, respectively. Prior studies that represented similar scenarios, however, have shown that estimated coefficients and standard errors are usually not biased, even with a sample size as low as 5 to 10 at Level 1 ^27, 28^, and with many independent variables and interaction terms ^29^.

Nevertheless, this study will use an MLM framework to assess the intervention effect based on a difference-in-difference analysis by computing differences between baseline and midline within each arm, and then test this difference between intervention and control arms. A fixed effects model will first be used to observe this effect of the intervention. Kebele and PHCU will then be included as random intercepts to account for clustering within these levels. The estimated variance for random effects will show the amount of variability in the outcome that is explained by the clusters (i.e., how much of institutional delivery uptake is attributed to a kebele). If the coefficient of the interaction term is significant with the significant model fit, we will observe significant program effects. The identical analyses will be conducted with 4+ANC visits as the outcome variable. Other covariates will be included by comparing goodness-of-fit statistics (e.g., AIC, BIC) to improve the model fit. Proper model specification and diagnostic tests will be conducted to enhance the robustness of the results. Phase 2 data analysis focus on comparing service utilization between intervention and control arms. Since we expect that study effectiveness will be stronger in Phase 2 with the implementation of enhanced solutions, we will additionally compare the study outcomes with baseline at Phase 1, controlling for potential key covariates including women’s education, parity, financial stability, geographical areas, and distance to health facilities.

### Qualitative data analysis

Translated transcripts of the in-depth interviews will be uploaded to a qualitative software package that will be used to facilitate data management and organization. After reading over a few of the interview scripts for each type of stakeholder, two analytical frameworks (one for pregnant women and one for health providers) will be developed using deductive and inductive approaches. The frameworks will describe themes, any sub-themes, codes, and illustrative quotes that would supplement each identified theme/sub-theme. Along with the interview data, field notes from monitoring visits, scheduled 4 times throughout implementation, will additionally reviewed since they can provide rich context for the implementation process itself. Field notes would capture the trajectory of implementation as to how stakeholders engage with the process over time and whether specific implementation strategies would effectively address any challenges arising from the field.

Codes related to implementation research outcomes will be pre-defined, but iterative coding will allow new ideas to emerge. Other themes related to change in social factors are expected to emerge from data and will be identified through a process of open coding. Throughout the coding process, memo writing will be conducted simultaneously. Memo writing refers to systematic and continuous note-making during analysis ^30^. While it has been primarily used in grounded theory, it is useful for a wide variety of qualitative methods because it provides researchers with an opportunity to critically reflect on how they view and treat data as well as any changes in decisions made ^31^. Iterative coding and memo writing would enable constant comparisons of the data and other reflections over the course of the analysis. Two to three coders will analyze the data to achieve inter-coder reliability. The qualitative data management and analysis will adhere to the Consolidated criteria for reporting qualitative research (COREQ) guideline ^32^.

### Ethics approval

This study was approved on October 5, 2023 by the Ethiopian Public Health Institute (EPHI), a governmental public health institution located in Addis Ababa, Ethiopia (EPHI-IRB-510-2023). The first phase of the study was also reviewed and approved by the Johns Hopkins Bloomberg School of Public Health Institutional Review Board (IRB00024473) on July 18, 2023, and the approval was extended for Phase 2 on July 16, 2024. In order to conduct HCD processes, we obtained ethical approval for public health practice by the Johns Hopkins Bloomberg School of Public Health Institutional Review Board (IRB00023366) on January 4, 2023. Any changes to study protocol will be communicated with these regulatory entities for approval immediately.

### Study dissemination

We will disseminate our work at conferences and peer-reviewed academic journals. We will also share our findings with program stakeholders, including government officials, health workers, and community workers.

### Participant consent and confidentiality

All participants will be interviewed at their convenient location to ensure privacy. Informed consent will be obtained in Amharic or Afaan Oromo by local data collectors who are externally hired and trained by the Center for Communication Programs (CCP) Ethiopia study team. Data collectors will read the consent document to participants, who will then give verbal consent. Participants under the age of 19 are required to obtain the permission of one parent or legal guardian. Obtaining verbal consent is considered appropriate given the low level of literacy and education among study participants. The ethics committees from the team’s primary institutions approved administrating verbal consent. All data from participants will be de-identified by the study team and stored in secure, password-protected computers accessible only by the study team and its affiliates.

## Discussions

The primary goal of the study is to determine whether, and to what extent, an intervention designed and implemented by adopting human-centered design principles can improve institutional delivery and 4+ ANC visits. While many interventions have been conducted to make an impact on these outcomes, two features of the current work are particularly noteworthy. First, the project adopts HCD principles throughout the life of the intervention, from the initial program design phase all the way to implementation and evaluation. A recent review indicates that most interventions identified as adopting HCD approaches do not do so in a holistic manner, limiting the HCD component predominantly to intervention design (Kang et al., under review). While adopting HCD principles throughout the project is often time consuming, requiring frequent reappraisals and adjustments, this project will provide some indications about the strengths (and limitations) of this approach to inform future projects.

The second innovative aspect of the current project is the extra effort we are expending on reaching women perceived to be most vulnerable. Given the high maternal mortality rate in Ethiopia, one can make the case that most women in the country are, in fact, vulnerable. Nevertheless, we make important distinctions between those who are vulnerable, writ large, and those whose individual, social, cultural, and environmental realities intersect in particularly devastating ways.

The rationale for this approach is our observation that, although maternal health outcomes are steadily improving in Ethiopia, there is a group of women who need support and approaches that are different from those being used to reach the larger majority, and we need interventions specifically tailored for this most-at-risk group. It is this belief that has resulted in a particular design of our study: we only include women whose vulnerability scores are moderate to high. It is likely that this approach requires extra resources, particularly if the most vulnerable women are geographically dispersed from each other and from the health services that they need to access. We suspect, however, that in the long run, these extra resources will turn out to be highly cost-effective, and we hope that dimensions will be designed to assess the cost-effectiveness and cost benefit of this approach.

The HCD solutions we are implementing in this project (e.g., delivering audio devices and supporting print materials) are, indeed, rather labor intensive and involve the adoption and distribution of new hardware. This intensity and the tailored approach, we anticipate, will be impactful among women who are exposed to the intervention. From a scale-up perspective, however, this may appear to be an approach that the government would be reluctant to adopt, given the required training and resources. We note that this approach is not meant to be scaled up for everyone; rather, the idea is that, for women whose life circumstances put them at the highest levels of risk, we need alternative approaches: ones that may be cost intensive but are, nevertheless, cost-effective. We hope to shed light on this tension between intervention intensity and its scale-up potential through the findings and recommendations that will emerge from this study.

### Study status

Our study is currently at the post-intervention evaluation stage of Phase 1. We completed the baseline data collection and intervention implementation. The midline (post-intervention) assessment of Phase 1 is ongoing.

## Data Availability

No data are associated with this article. OSF: Enhancing maternal health service utilization among highly vulnerable pregnant women through a human-centered design process: Study protocol for a quasi-experimental study in Oromia, Ethiopia. https://doi.org/10.17605/osf.io/cmnps ^33^. This project contains the following underlying data: Appendix 1_Screening Tool. Pregnancy and vulnerability screening tool. Appendix 2_Survey questionnaire. Baseline and midline survey guide. Appendix 3_Qualitative guide Phase 1_Pregnant women. Qualitative questionnaire for pregnant women. Appendix 4-6_Qualitative guide Phase 1_Providers. Qualitative questionnaire for health care providers. OSF: SPIRIT Checklist for “Enhancing maternal health service utilization among highly vulnerable pregnant women through a human-centered design process: Study protocol for a quasi-experimental study in Oromia, Ethiopia.” https://doi.org/10.17605/osf.io/cmnps ^33^. Data are available under the terms of the Creative Commons Zero "No rights reserved" data waiver (CC0 1.0 Public domain dedication).
