# Developing and evaluating human-centered design solutions for enhancing maternal health service utilization among vulnerable pregnant women in Oromia, Ethiopia: Study protocol for a quasi-experimental study

**DOI:** 10.12688/gatesopenres.16277.3

**Published:** 2025-02-21

**Authors:** Bee-Ah Kang, Habtamu Tamene, Yihunie Lakew, Daryl Stephens, Rajiv Rimal

**Affiliations:** 1 Department of Health, Behavior and Society, Johns Hopkins University Bloomberg School of Public Health, 624 N. Broadway, Baltimore, MD, 21215, USA; 2 Center for Communication Programs, Johns Hopkins Bloomberg School of Public Health, Africa Avenue (Bole Road), Dembel City Center 10th Floor, P.O Box: 26171, Addis Ababa, 1000, Ethiopia

**Keywords:** maternal and child health, vulnerability, human-centered design, research protocol

## Abstract

**Background:**

Disproportionate uptake of and access to maternal and child health services remain significant challenges across and within countries. Differing geographic, economic, environmental, and social factors contribute to varying degrees of vulnerabilities among individuals, which manifest as disparities in maternal and newborn health outcomes. Designing solutions according to need is vital to improve maternal and child health outcomes. In this paper, we describe our study protocol on developing and evaluating the effectiveness of human-centered design (HCD) solutions to improve maternal health service uptake among vulnerable pregnant women in rural areas of Ethiopia.

**Methods:**

The study has two distinct phases. In Phase 1, HCD solutions were developed through co-design workshops with vulnerable pregnant women and key stakeholders. Final solutions included home visit education, audio programs promoting couple discussion, and print materials, implemented in collaboration with community health workers and health officers. A community-based, quasi-experimental, mixed-method study design was used to assess differences between intervention and control arms. A panel sample was enrolled after screening for pregnancy and vulnerability level and surveyed at baseline and midline in Phase 1. Phase 2 adopts an identical design approach with a focus on refining Phase 1 solutions. Newly recruited pregnant women will receive refined solutions for six months, which will be evaluated using post-only end-line surveys and in-depth interviews.

**Conclusions and Implications:**

Our sequential approach to evaluating initial solutions, which in turn will inform the enhancement of solutions, will provide practical insights into how solutions are accepted among vulnerable women and how they can be better integrated into women’s lives and health systems. This will inform equity-focused practice and policies targeting populations experiencing greater barriers to accessing care and provide insights into system strengthening in rural areas. Our findings will be disseminated to the Ethiopian Ministry of Health and its partners to inform large-scale implementation at the national level.

## Introduction

Reducing maternal mortality ratio has been one of the major priorities on the global health agenda. With the launch of the Sustainable Development Goals in 2015, the World Health Organization (WHO) and partners reached a consensus on endorsing Target 3.1 that aims to reduce the global MMR to less than 70 per 100,000 livebirths by 2030. The target has shown a steady progress with its 34.3% reduction between 2000 and 2020 ^1^. However, such aggregated outcomes in health indicators often obscure disparities across countries and within sub-regions.

Ethiopia has shown steady improvements in many maternal and child health indicators over the past two decades. In 2000, the maternal mortality ratio was 871 deaths per 100,000 livebirths, which declined to 412 in 2016 ^2^. Between 2005 and 2019, under-5 mortality also decreased by 52% ^3^. Despite this substantial progress, Ethiopia faces persistent health challenges. The maternal mortality ratio in-country accounts for 3.6% of global maternal mortalities ^4^. Twenty-five percent of female deaths from the 2016 data were found to be from pregnancy-related causes ^2^, and the neonatal mortality rate also increased by four points per 1,000 livebirths between 2016 and 2019 ^3^.

To achieve faster and more equitable improvements in maternal and child health outcomes, the government of Ethiopia has implemented a three-tiered health system with an intensive Health Extension Program, which takes a community-based approach to improving health knowledge, health-related skills, and access to primary healthcare ^5^. In addition, the Health System Transformation Plan (HSTP-II) is being implemented from 2020 to 2025 with an overall objective to improve the population’s health status by accelerating progress towards universal health coverage, protecting populations during health emergencies, reducing home delivery across woredas (districts), and improving health system responsiveness to the needs of target populations and communities ^6^. The HSTP-II has a target to reduce the maternal mortality ratio to 279 per 100,000 live births and under-5 and neonatal mortalities to 44 and 21 per 1,000 live births, respectively ^6^. Under these national goals, providing pregnant women with essential maternal health services, including institutional delivery (i.e., childbirth in health facilities attended by skilled birth attendants), antenatal care (ANC), and iron and folic acid supplementation, has been a key strategy for optimizing maternal and child health outcomes in Ethiopia. These maternal health services can significantly reduce the risk of maternal, perinatal and neonatal mortality in low- and middle-income countries and alleviate health disparities worldwide ^7– 9^.

Nevertheless, there remains an urgent need to address disproportionate uptake of and access to maternal health services in Ethiopia. Taking institutional delivery coverage as an example, women’s use of health facilities for childbirth greatly varies by region and sociodemographic characteristics. With the national average being approximately 48%, the percentage of institutional delivery use ranges from 23.3% in Somali Region to 94.8% in Addis Ababa, the capital city ^3^. Also, 79% of women in the lowest wealth quintile delivered a baby at home; the corresponding figure for women in the highest wealth quintile was 14%. Parity is also associated with home delivery: women having six or more births are more likely to deliver at home, compared to those delivering for the first time.

Prior evidence suggests that women’s use of maternal health services is shaped by multiple factors at the socio-ecological continuum. Barriers to maternal health service uptake among pregnant women reside at multiple levels, including at the individual level (having poor knowledge of obstetric complications and danger signs, low risk perceptions, high parity, and no birth preparedness plans) ^10– 12^, social level (social and gender norms, support from family, decision-making power) ^11, 13, 14^, cultural level (respect for elders, perception about pregnancy-related matters as women’s privacy, relationship with traditional birth attendants in the community) ^3, 15– 17^, structural level (distance to health facilities, transportation and road infrastructure, costs for traveling, delivery cost) ^11, 15, 18^, and those that are service-related (lack of services that respect women’s privacy and preferences, disrespectful treatment by health workers, and staff and medical supply shortages) ^10, 15, 19, 20^.

Understanding vulnerabilities of key target populations has important implications for developing and implementing tailored programs and policies ^21^. Nevertheless, less attention has been paid to how vulnerability is defined, conceptualized and operationalized in the realm of maternal health service coverage ^22^. Indeed, despite remarkable improvements in disease-focused service coverage, global health programs have been highlighted as neglecting inequity by not reaching the most disadvantaged population, straining weak local health systems, and distorting local health priorities and agendas ^23, 24^. Our project is based on the assumption that accurate and comprehensive assessment of vulnerability in maternal service uptake can help identify vulnerable pregnant women with the lowest likelihood of accessing necessary health services, which will subsequently help generate evidence and provide guidance on effective policies and interventions. Therefore, identifying the most vulnerable pregnant women and engaging them in program development would enhance the applicability, viability, and effectiveness of an intervention. Adopting user-oriented strategies, our study attempts to render vulnerable pregnant women an opportunity to take ownership of their problems and participate in generating solutions with the study team to cater to their unique needs, challenges, and hopes.

Given the complex interplay of factors which prevent or hinder access to maternal health services, and with special attention to the social and economic factors which underpin vulnerability in this context, we chose to adopt a human-centered design (HCD) approach in the development of the solutions. Prior interventions effectively developed HCD-based health solutions, taking account of multiple factors, including decision-making power, gender roles, and social norms ^25, 26^. HCD leverages creative thinking through an iterative process and has proven to be a meaningful tool in program and solution design ^27, 28^. HCD can offer a novel approach to solution design through putting key stakeholders, in this case pregnant women and the network of providers and volunteers who offer care during pregnancy, at the center of the process to find solutions that are most relevant to their daily lives and circumstances. Further, the iterative nature of HCD allows for the refinement of solutions along multiple axes, including feasibility, sustainability, and ability to be enmeshed in pre-existing health systems.

Furthermore, we argue for the need for conducting implementation research, given that determining *true* program effectiveness necessitates evidence on how an intervention fits in “real world” settings beyond a controlled study environment. Implementation research is distinguished from monitoring or process evaluation efforts due to its focused goals to explore 1) a program’s likelihood of being adopted and scaled up in real-world settings, 2) contextual factors that may facilitate or hinder implementation, 3) implementation strategies (rather than intervention strategies), 4) and the engagement of stakeholders to achieve successful implementation ^25^. Well-designed implementation research provides an insight into whether an intervention would likely be integrated into its target environment, and more broadly, into health systems beyond study duration and scope. Implementation research also generates evidence that helps policy makers and implementers foresee challenges, contributing to saving costs and resources for future programs. Although there is no scientific consensus on what elements and principles are necessary for conducting implementation research, the WHO guide ^26^ encourages researchers to outline implementation research questions, use a framework that describes implementation research outcomes, and select a study design and methods that are most pertinent to understand how implementation occurs in a given context.

### Study objectives

The objective of this study is to develop and evaluate the effectiveness of an HCD intervention package through a two-phased sequential study design. Our objective tailored to intervention development is to co-design solutions with vulnerable pregnant women and key stakeholders to improve maternal health service (institutional delivery and ANC) uptake in rural Oromia region in Ethiopia based on 5-step design processes. In Phase 1, HCD solutions (home visit education, audio programs, and print materials) were finalized in January 2024 and implemented until May 2024. Our objective in Phase 2 is to refine the solutions to optimize scalability and ownership in the health system, in turn to ensure service uptake among pregnant women. Our evaluation objectives across the two phases are:

1. To assess whether changes in institutional delivery and ANC visits among women in the intervention arm are significantly greater than corresponding changes in the control arm.
2. To explore how social and behavioral factors, including social norms, gender norms, women’s empowerment, and spousal dynamics, are associated with maternal health service uptake.
3. To understand the extent to which the intervention meets key implementation research outcomes, including fidelity, acceptability, feasibility, scalability, and sustainability, among pregnant women and health workers in the intervention arm.

## Methods

### Study setting

**Figure 1.**
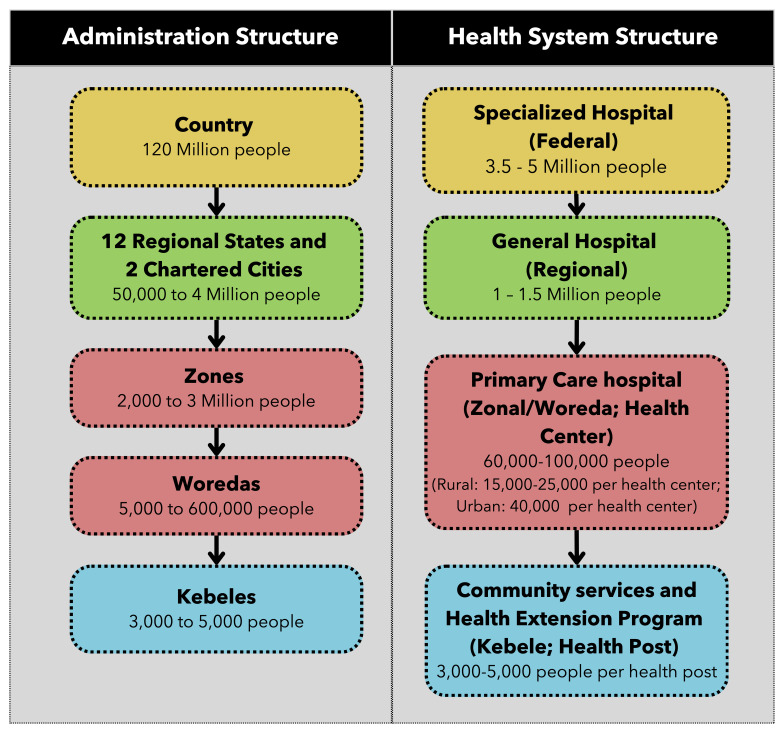
Administration and health system structure in Ethiopia, adapted from the Federal Ministry of Health (FMOH).

Administratively, Ethiopia is divided into 12 regions and two administrative cities, with regions further subdivided into zones, woredas, and kebeles (the lowest administrative unit), as described in Figure 1. Enumeration areas are created by the Ethiopian Statistical Services for the purposes of population and housing census-taking to serve as clusters that typically contain about 750 to 1000 people, with each kebele possibly containing multiple enumeration areas.

Ethiopia's health system consists of three tiers: the primary tier includes one primary hospital, five health centers, and 25 health posts serving about 100,000 people; the secondary tier comprises general hospitals serving 1 to 1.5 million people; and the tertiary tier includes specialized hospitals serving 3.5 to 5 million people.

Our study sites are in Oromia, the most populous region in Ethiopia. Within this region, Siraro and Shalla woredas were selected as intervention arms, and Gera and Shebe woredas as control arms. The study sites were not randomly selected due to the on-going security issues in Ethiopia. The distance of woredas from major regional towns, maternal and child health service utilization (obtained from the national Health Management Information System data), and major livelihoods of the target study population were taken into consideration in the selection of intervention and control woredas. Two woredas (one nearby and one remote from towns) with similar health service uptake and predominantly agricultural livelihoods were assigned as the intervention group. The same strategies were used to select the control woredas. 

### Study design

A community-based quasi-experimental study design was chosen to assess differences in key variables between the intervention and control arms. We chose a quasi-experimental design for several reasons. We lacked information about clear randomization unit given the characteristics of individuals or clusters (woredas), and it was likely that many woredas in Oromia region are not homogenous towards our outcomes of interest. Another reason was due to resource constraints, as we did not have a sufficient number of woredas in our study to conduct a pure randomized trial design. The study included two waves of data collection from a panel of recruited pregnant women at Phase 1. We attempt to leverage Phase 1 findings to inform the development of study tools and identify factors affecting implementation to provide insights into the improvement our intervention at Phase 2. This study is registered at ClinicalTrial.gov (NCT05907720).

In Phase 1, we conducted the baseline survey with 470 pregnant women enrolled in intervention and control arms in January 2024. After the baseline survey, we implemented a set of HCD prototype solutions in the intervention arm only, keeping the usual standard of care intact in the control arm. After the 4-month period of HCD solution implementation, we conducted the midline survey among the same women in the panel from both the intervention and control arms. In the midline survey, we interviewed 453 pregnant women, with 17 pregnant women lost to follow-up. The midline assessment also included in-depth interviews with purposively selected pregnant women from the panel and health workers who engaged in implementation.

In Phase 2, we will conduct a post-only study with a new sample of vulnerable pregnant women who will be recruited after the implementation of refined HCD solutions. Since the focus is on enhancing implementation feasibility, scalability, and health system ownership of Phase 1 solutions, which have already shown significant improvements in institutional delivery, we believe a post-only assessment is sufficient to observe consistent effectiveness. 

**Figure 2.**
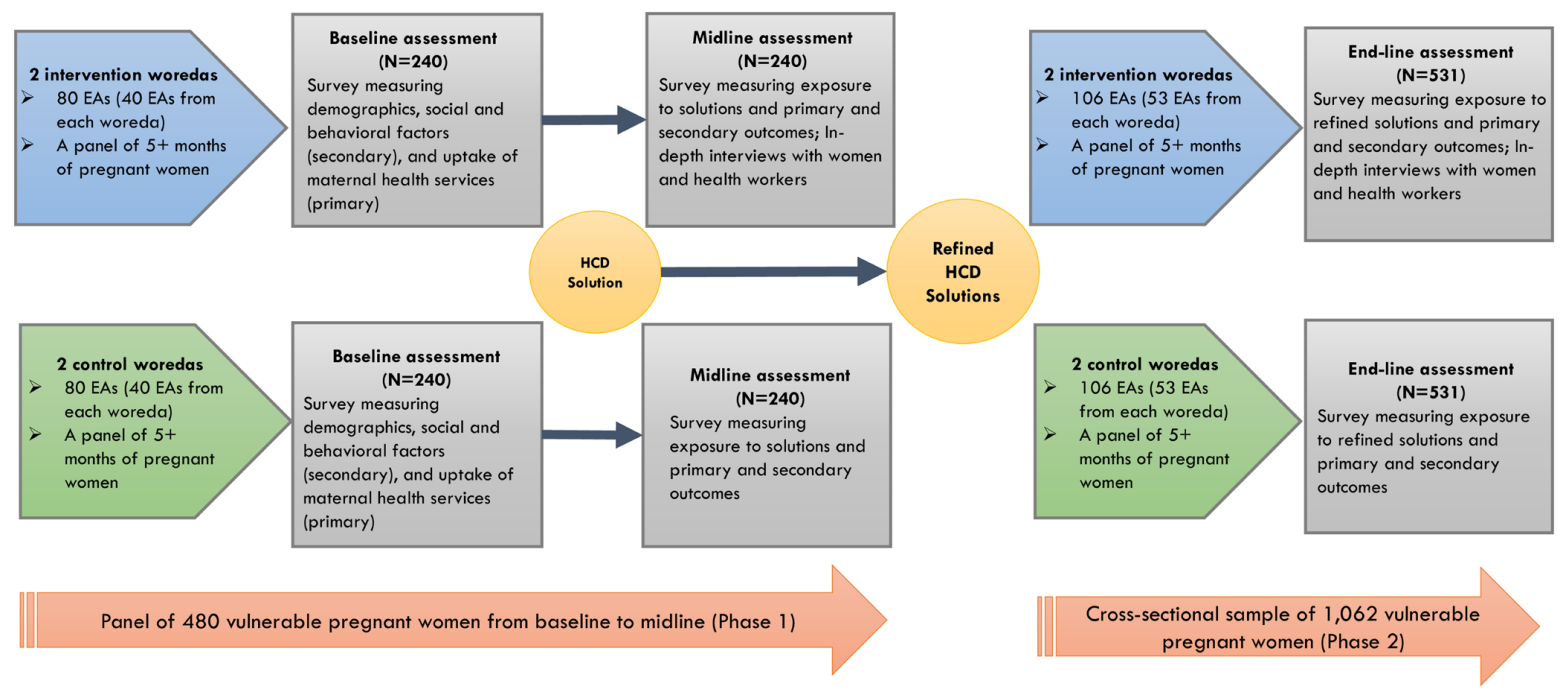
Quasi-experimental evaluation study among vulnerable pregnant women.

We will compare outcomes between the intervention and control arms at end-line, and the end-line findings will also be compared with the baseline to identify whether refined solutions bring greater impact than our initial solutions did in Phase 1. Thus, our overall study design includes three waves of data points: baseline and midline assessments in Phase 1 and end-line assessment in Phase 2. The study design is further described in Figure 2.

### Sample

Our primary target participants are vulnerable pregnant women aged 15–49. For consent administration, we segmented adult pregnant women aged 19 and above, pregnant adolescent girls aged 15–18, and service providers. Service providers, such as midwives, health extension workers and health development armies who had engaged in the study were recruited for qualitative assessment at midline (Phase 1) and will be enrolled in end-line (Phase 2). Table 1 below summarizes inclusion and exclusion criteria for each sample population.

**Table 1.** Summary of inclusion and exclusion criteria of study sample.

Respondent type	Inclusion criteria	Exclusion criteria
Pregnant women	Women in their 5th or higher months of pregnancy; meet the high vulnerability criteria.	Pregnant women who are less than 5 months pregnant; meet the low vulnerability criteria.
Women who delivered recently (Midline and end-line only)	Recently delivered women included in the panel for baseline assessments during Phase 1; those who have received HCD solutions during Phase 2; meet the high vulnerability criteria.	Recently delivered women who were not included in the panel; did not have exposure to HCD solutions; meet the low vulnerability criteria.
Heath Extension Workers	Who work in health promotion and essential maternal health services in heath posts.	Who have worked in this position less than 6 months in the intervention arm.
Health Development Armies	Community volunteers who work on early screening and health promotion activities for pregnant women.	Who have worked in this position less than 6 months in the intervention arm.
Head of woreda health office	Head/Vice Head of the woreda Health Office who help facilitate implementation of the project	Who have worked in this position less than 6 months in the intervention arm.

### Sample size calculation

For Phase 1, we considered institutional delivery (a major outcome variable) as a basis for the sample size calculation. To estimate sample size, we took account of our longitudinal design that measures the same subjects before and after the intervention and assumed an intervention effect of 9% in improving institutional delivery beyond the current 48% ^3^. Assuming a p-value of 0.05, interclass correlation coefficient (ICC) of 0.009, a power of 80%, a total of 240 pregnant women from the intervention arm and another 240 pregnant women from the control arm are needed, for a total sample size of 480 pregnant women in each study phase. The assumptions of sample size also took into account feasibility and sample characteristics. The number of enumeration areas in the first phase was 80 per study arm, and approximately 5 women were expected to be eligible in each enumeration area. Based on the cross-sectional study design in Phase 2, we assumed 9% detectable difference between the study arms with an 80% power, which led to a sample of 531 for each study arm, taking account of 10% non-response rate. Approximately 106 enumeration areas will be reached from given the number of eligible women in each area. Both intervention and control arms will have an equal sample size in each phase, having 1:1 ratio of women between the intervention and control arms. As such, the total number of women to recruit across study arms in Phase 1 will be 1062.

For qualitative assessments, we use the same approach in Phase 1 and 2. An equal number of health workers (n= 32) are interviewed at mid-line (post-intervention of Phase 1) and end-line (post-intervention of Phase 2). The interviews with health workers explore the feasibility, acceptability, and scalability of the prototype solutions along with perceived program effects. The interviewees selected for Phase 2 may be the same service providers recruited from Phase 1 depending on their availability and engagement in the program. We have chosen 32 as the target sample size based on our best estimate about achieving data saturation. In each phase, we will also interview 20 pregnant women from the panel who recently delivered and were exposed to our HCD solutions. The qualitative interviews will be conducted only in the intervention sites.

### Maternal Vulnerability Segmentation Tool

A vulnerability screening tool was developed to identify pregnant women with heightened vulnerabilities. We performed logistic regression analyses using the 2019 Ethiopian Demographic and Health Survey to identify factors associated with ANC attendance and institutional delivery. Next, a multivariate cluster analysis (K-medoids) was conducted to evaluate the tool’s ability to classify pregnant women as having low, moderate, or high vulnerability. The initial validation of the tool was conducted through expert reviews and group discussions with healthcare workers. Feedback from these stakeholders helped evaluate whether the screening items were comprehensive and relevant to women living in the study areas. The tool was then pretested with the target group of pregnant women to ensure its clarity and applicability. This process allowed us to refine the questions and incorporate illustrations to enhance understanding among rural communities.

The final screening tool included 20 items assessing individual, social, structural, and service-level determinants. Women who scored less than 12 were classified as having low vulnerability, those scoring 12–15 as moderate, and those scoring greater than 16 as highly vulnerable. Thus, an aggregate score of 12 or more indicated moderate to high vulnerability. These cutoffs were determined as ANC and health facility delivery were lower among pregnant women with moderate and high vulnerability scores compared to those with low vulnerability scores.

### Recruitment

After obtaining a list of all interested women from health extension workers and health development armies, data collectors visit the women in person to screen their pregnancy status and vulnerability level to assess their eligibility. With the woman’s verbal consent, the data collector administers the pregnancy screening tool to identify her pregnancy status. Only women at 5+ months of pregnancy and aged 15–49 are considered eligible. If eligible, she is screened for her vulnerability level, assessed through the vulnerability screening tool. Those considered to be moderately to highly vulnerable are eligible and entered into the sampling frame. Women who score at least 9 out of the maximum 20 vulnerability screening questions will be identified as a study participant. All responses to the screening questions will be entered into the data collectors’ mobile phone application that automatically determines pregnant women’s eligibility during the visit. 

All eligible women are entered into our sampling frame. After listing eligible pregnant women, we aim to recruit approximately three women per enumeration area to achieve our target sample size. If an enumeration area has five or fewer eligible women, they are all contacted for recruitment. If there are more pregnant women than the minimum required number of pregnant women, we randomly select the required number. If a woman is not interested or available for participation, we contact the next randomly selected woman for recruitment. Recruitment continues until our sample size is reached, expanding to other enumeration areas if necessary. Recruitment in Phase 1 was completed in this way, and Phase 2 recruitment will follow this procedure.

For the qualitative assessment, recently delivered women who receive our intervention and health workers in the intervention sites are purposively recruited. Purposive selection of the sample allows us to understand the experiences of engaging in our implementation process among diverse individuals. For women, the selection criteria include: 1) residing in Siraro and Shalla woredas, 2) being exposed to the HCD solutions during their last pregnancy, and 3) having delivered after receiving the solutions. To further obtain diverse and in-depth experiences among our sample, we prioritize recruiting women who are in earlier gestational ages (5 ^th^ month or earlier) at the time of recruitment (before intervention), as those in late pregnancy terms may not be fully exposed to our solutions. Also, both women who delivered in health facilities and at home are contacted for recruitment to capture a broad range of experiences. We also attempt to ensure diversity in kebeles and primary health care units in the selected sample to ascertain potential differences. The criteria for recruiting health workers include: 1) those working in Siraro and Shalla woredas, 2) serving as a health extension worker, health development army, primary health care unit (PHCU) staff in the woreda, or health officials such as woreda health leaders, and 3) being engaged in the implementation of the HCD solutions. Trained data collectors administer consent and interview with eligible pregnant women at their homes and with health workers at their workplaces (e.g., local health center). We will use the same sampling approach for both midline and end-line qualitative assessments. 

### Data collection and measurement

***Survey interview*.** All participants from Phases 1 and 2 undergo a one-on-one survey interview to assess sociodemographic information, psychological factors, social and structural factors, and maternal health service utilization. A structured interview is administered by a local data collector in the local language. The survey captures the self-reported place of the most recent childbirth along with uptake and frequency of ANC visits during the most recent pregnancy. Secondary outcomes include knowledge, attitudes, and perceptions about maternal health service utilization, social norms, gender norms, spousal dynamics, decision-making, and community and structural factors. Midline (post-intervention in Phase 1) and end-line (post-intervention in Phase 2) assessments additionally include questions measuring program exposure.

***Qualitative interview*.** Purposively selected women from the study participants (n=20) along with health workers (n=32) engaged in the program participate in an in-depth interview. Given the 4-month duration of program implementation among pregnant women in their 5 ^th^ or higher months of pregnancy, interviews are administered to recently delivered women inquiring about their perceived effects of the intervention on the place of childbirth and ANC uptake during pregnancy. Additionally, the interview guides for women and health workers contain questions around key implementation research outcomes, including acceptability and feasibility, to identify any similarities and differences arising across different types of interview participants.

### Intervention

***HCD workshop procedure*.** In Phase 1, prior to the HCD process, an extensive desk review and secondary data analysis were conducted to identify key drivers of vulnerability affecting uptakes of maternal and child health services (ANC and institutional delivery). Vulnerability driving factors, such as women's illiteracy, distance from health facilities, high parity, decision-making power, exposure to media, and lack of household assets were identified as major barriers to maternal health service use. Based on these factors, a vulnerability screening tool was developed to identify the target population of rural vulnerable pregnant women. Couples who met three or more of the vulnerability criteria among those driving factors (e.g., living over 30 minutes from a health facility, being illiterate, having poor housing conditions, and lacking a mobile phone) were identified. From a desk review and evidence synthesis of secondary data, two initial design challenges focusing on ANC and institutional delivery were prioritized: 1) reimagining how to support pregnant women to attend ANC, and 2) reimagining how to support pregnant women to deliver in a health center. Then, an iterative five-phase design thinking process was implemented, including empathy, define, ideate, prototype, and testing that provides a flexible framework for co-creating solutions with community members and heath care stakeholders. 

The process involved a series of co-creation workshops in two rounds with diverse stakeholders. The initial round of workshops included pregnant women and husbands, organized as separate workshops to ensure open discussion. A total of eight teams (four teams of pregnant women and four teams of husbands) were organized in the two implementation woredas. A design team constituted up to six participants per concurrent session. Four teams focused on the design challenge on ANC, while the remaining focused on institutional delivery. These teams engaged in the empathy, design, and ideate phases of the HCD process. Subsequently, another set of workshops was conducted with healthcare providers (health development army, health extension workers, midwives/nurses, PHCU directors and woreda maternal and child health coordinators) organized into eight different teams: four on the first design challenge and four on the second. The second round of workshops was led by trained design workshop facilitators, building on the first-round outputs to ensure key insights from the teams of pregnant women and husbands were not lost. The mid-fidelity prototype solutions were tested with vulnerable pregnant women, husbands and community health workers to assess feasibility and acceptability. A total of 204 participants engaged in workshops and prototype testing. A core team of senior design staff further synthesized the outputs of the first and second round workshops. Insights around limited household support for pregnant women, low awareness on importance of healthcare services, demotivated healthcare providers, and poor service quality were harvested from these workshops.

***Development of HCD solutions*.** In the prototyping phase, our team prioritized potential prototype solutions based on an impact and feasibility matrix. The matrix served as a grading system to narrow down prototype solutions from those that were initially ideated among workshop participants based on the two dimensions: perceived impact (low or high) of solutions on institutional and ANC uptake and perceived feasibility (less or more likely to be implemented) pertaining to resource constraints. For example, while some solutions addressing structural-level factors, including transportation, were perceived as impactful, they were eliminated due to limited resources to address them.

Ultimately, three solutions were selected: a self-paced, audio-based program on ANC and health facility delivery to promote couple discussion on these issues, visual print materials reinforcing the audio content on ANC and institutional delivery, and home visits by health development armies (community health volunteers) to deliver these materials and provide interpersonal education to couples. Our final solutions primarily addressed individual and social-level factors, such as perceptions, knowledge, and social and gender norms, while also partially influencing service-level factors by improving service providers' perceptions and engagement.

The study team developed two pre-recorded audio programs containing real stories of pregnant women in the local language of the target area. The first audio session focuses on the importance of ANC through the narration of a couple's story, highlighting their communication and the husband's support in household tasks, as well as accompanying the wife to ANC visits. The second audio session focuses on the importance of institutional delivery from a story of a pregnant woman. It describes her first childbirth at home, which was a painful and difficult experience. The story then progresses to her second childbirth experience at a health facility, where she was well-prepared and supported by her husband. The two visual print materials (pamphlets) included graphics and text emphasizing behavioral action points that reinforced the audio messages. These prototypes were tested through focus group discussions with pregnant women, their husbands, healthcare providers and community volunteers, and then refined based on user feedback. Most responses were positive, with participants expressing appreciation for the relatable storytelling and the ease of engaging with the content at their own pace. Minor suggestions were received, primarily concerning the clarity of certain audio elements. Based on this feedback, minor adjustments were made to enhance the prototype’s usability and appeal. All five of the design thinking stages were implemented in the two study sites (Shalla and Siraro) of Oromia regional state.

For Phase 2, we aim to leverage implementation research data from Phase 1 to finalize our HCD plans and solutions. Learnings from Phase I indicated that the solutions significantly increased institutional delivery rates among vulnerable pregnant women. However, the production costs of the solutions, the intensity of implementation requiring close follow-up and supervision at health facilities, and challenges in ensuring health system ownership for scalability and sustainability were identified as major bottlenecks. Thus, HCD in Phase 2 will focus on the new design challenge: reimagining the couple communication-based interventions to enhance scalability and health system ownership, with the goal of increasing institutional delivery rates among vulnerable pregnant women.

We will apply the same five-stage HCD process in Phase 2. Since Phase 2 will focus on refining and strengthening the existing solutions, some of the HCD activities (e.g., “How Might We” statements and ideation) are expected to center around strategies for improving the materials and implementation delivery mechanisms through which scalability and health system ownership will be ensured. The HCD solutions will be tested with two target populations (pregnant women and implementers).

### Implementation plan

As part of the implementation of the interventions in Phase 1, the study team conducted an orientation training for health extensions workers and health development armies (known as Hadha Gare locally) to equip them with the skills needed to identify vulnerable pregnant women using a vulnerability screening tool and to effectively implement the solutions. The prototype solutions were delivered by the trained health development army with the help of health extension workers for a duration of four months from February to May 2024.

Prior to implementation, the health development army identified vulnerable pregnant women within their catchment area. They then scheduled an appointment to facilitate family discussions about the prototype solutions. The audio programs and print materials were intended to be delivered throughout five home visits by the health development army. For women whose gestational age was high (8–9 months), we aimed to provide audio and print materials on institutional delivery through three home visits.

In the first visit, the health development army provided the household with an audio device containing the pre-recorded ANC program and gave instructions on how to engage in couple communication around the program. After two to three days, the health development army revisited the household to inquire about questions or concerns from the couple, collected the device, and provided a print material to reinforce the audio content. The health development army then conducted a third visit to gather feedback from the couple and provided the second audio device that contained a story of institutional delivery. At the fourth visit, the health development army collected the second device and provided the second print material to reinforce institutional delivery messages from the audio program. Finally, the health development army conducted the final visit, encouraging the pregnant woman to utilize health services until she delivers and addressing remaining concerns among the couple. All of the home visits required the husbands’ presence given that the solutions were designed to facilitate couple communication, which would in turn optimize male involvement in maternal health service potentially through shifting social and gender norms. However, if the husband was not available for long durations during the implementation period, the health development army facilitated discussions with pregnant women and other family members.

To ensure seamless implementation of the interventions, the study team conducted close monitoring of activities that included site review meetings with implementing partners at the PHCU level and on-site supportive supervision to identify gaps, provide technical support, and address any identified implementation challenges. Key implementation outcome indicators, such as the number of pregnant women reached with ANC and institutional delivery audio programs and print materials, were regularly collected through the KoboCollect mobile platform.

### Data analysis plan

***Quantitative data analysis*.** First, we will conduct a series of bivariate tests, including chi-square and t-tests, across treatment and control arms based on baseline and endline data. The purpose of conducting bivariate tests is to identify differences between the study arms for each potential confounding factors, given the possibility of quasi-experimental design not ensuring baseline matching between intervention and control arms. If any differences are observed, they will be controlled for in subsequent regression analyses. We will also identify types of missing data to determine whether missingness occurs in a systematic manner, which will help us determine the appropriate remedial measure to adopt.

Since this project will conduct various implementation activities at the PHCU level, the analysis will take account of clustering at the level. Further, because *kebeles* (akin to villages) are clustered within PHCUs, we will also consider clustering at the *kebele* level. Given the nested nature of this study, multi-level modeling is considered appropriate because it provides both cluster-specific and population-averaged estimates simultaneously by defining both random effects and fixed effects, respectively. Prior studies that represented similar scenarios, however, have shown that estimated coefficients and standard errors are usually not biased, even with a sample size as low as 5 to 10 at Level 1 ^27, 28^, and with many independent variables and interaction terms ^29^.

Nevertheless, this study will use a multi-level modeling framework to assess the intervention effect based on a difference-in-difference analysis by computing differences between baseline and midline within each arm, and then test this difference between intervention and control arms. A fixed effects model will first be used to observe this effect of the intervention. Kebele and PHCU will then be included as random intercepts to account for clustering within these levels. The estimated variance for random effects will show the amount of variability in the outcome that is explained by the clusters (i.e., how much of institutional delivery uptake is attributed to a kebele). If the coefficient of the interaction term is significant with the significant model fit, we will observe significant program effects. The identical analyses will be conducted with 4+ANC visits as the outcome variable. Other covariates will be included by comparing goodness-of-fit statistics (e.g., AIC, BIC) to improve the model fit. Proper model specification and diagnostic tests will be conducted to enhance the robustness of the results. Phase 2 data analysis will focus on comparing service utilization between intervention and control arms. Since we expect that study effectiveness will be stronger in Phase 2 with the implementation of enhanced solutions, we will also compare the study outcomes with baseline at Phase 1.

### Qualitative data analysis

Translated transcripts of the in-depth interviews will be uploaded to Dedoose ^30^ (Version 9.2.22), a qualitative analysis software that will be used to facilitate data management and organization. After reading over a few of the interview scripts for each type of stakeholder, an analytical framework will be developed using deductive and inductive approaches. The framework will describe themes, sub-themes, codes, sub-codes and illustrative quotes that would supplement each identified theme/sub-theme. Along with the interview data, field notes from monitoring visits (scheduled 4 times throughout implementation) will be additionally reviewed since they can provide rich context for the implementation process itself. Field notes would capture the trajectory of implementation as to how stakeholders engage with the process over time and whether specific implementation strategies would effectively address any challenges arising from the field.

Codes related to implementation research outcomes will be pre-defined, but iterative coding will allow new ideas to emerge. Other themes related to change in social factors are expected to emerge from the data and will be identified through a process of open coding. Throughout the coding process, memo writing will be conducted simultaneously. Memo writing refers to systematic and continuous note-making during analysis ^31^. While it has been primarily used in grounded theory, it is useful for a wide variety of qualitative methods because it provides researchers with an opportunity to critically reflect on how they view and treat data as well as any changes in decisions made ^32^. Iterative coding and memo writing will enable constant comparisons of the data and other reflections over the course of the analysis. Two to three coders will analyze the same data to achieve consensus in the beginning, then analyze the rest of the transcripts individually. The data analysis team will meet regularly to ensure consistency in coding. The qualitative data management and analysis will adhere to the Consolidated criteria for reporting qualitative research (COREQ) guidelines ^33^.

### Ethics approval

This study was approved on October 5, 2023 by the Ethiopian Public Health Institute (EPHI), a governmental public health institution located in Addis Ababa, Ethiopia (EPHI-IRB-510-2023). The first phase of the study was also reviewed and approved by the Johns Hopkins Bloomberg School of Public Health Institutional Review Board (IRB00024473) on July 18, 2023, and the approval was extended for Phase 2 on July 16, 2024. In order to conduct HCD processes, we obtained ethical approval for public health practice by the Johns Hopkins Bloomberg School of Public Health Institutional Review Board (IRB00023366) on January 4, 2023. Any changes to study protocol will be communicated with these regulatory entities for approval immediately.

All ethical requirements were adhered to during Phase 1, including administering recruitment scripts, obtaining consent, and ensuring the privacy of study participants as well as the confidentiality of the information collected.

### Study dissemination

We will disseminate our work at conferences and peer-reviewed academic journals. We will also share our findings with program stakeholders, including government officials, health workers, and community workers.

### Participant consent and confidentiality

All participants will be interviewed at their convenient location to ensure privacy. Informed consent will be obtained in Amharic or Afaan Oromo by local data collectors who are externally hired and trained by the Center for Communication Programs (CCP) Ethiopia study team. Data collectors will read the consent document to participants, who will then give verbal consent. Participants under the age of 19 are required to obtain the permission of one parent or legal guardian. Obtaining verbal consent is considered appropriate given the low level of literacy and education among study participants. The ethics committees from the team’s primary institutions approved administrating verbal consent. All data from participants will be de-identified by the study team and stored in secure, password-protected computers accessible only by the study team and its affiliates.

## Discussions

The primary goal of the study is to determine whether, and to what extent, an intervention designed and implemented by adopting HCD principles can improve institutional delivery and ANC visits. While many interventions have been conducted to make an impact on these outcomes, two features of the current work are particularly noteworthy. First, the project adopts HCD principles throughout the life of the intervention, from the initial program design phase all the way to implementation and evaluation. A recent review indicates that most interventions identified as adopting HCD approaches do not do so in a holistic manner, limiting the HCD component predominantly to intervention design (Kang *et al.*, under review). While adopting HCD principles throughout the project is often time consuming, requiring frequent reappraisals and adjustments, this project will provide some indications about the strengths (and limitations) of this approach to inform future projects.

The second innovative aspect of the current project is the extra effort we are expending on reaching women perceived to be most vulnerable. Given the high maternal mortality rate in Ethiopia, one can make the case that most women in the country are, in fact, vulnerable. Nevertheless, we make important distinctions between those who are vulnerable, writ large, and those whose individual, social, cultural, and environmental realities intersect in particularly devastating ways.

The rationale for this approach is our observation that, although maternal health outcomes are steadily improving in Ethiopia, there is a group of women who needs support and approaches that are different from those being used to reach the larger majority, and we need interventions specifically tailored for this most-at-risk group. It is this belief that has resulted in a particular design of our study: we only include women whose vulnerability scores are moderate to high. It is likely that this approach requires extra resources, particularly if the most vulnerable women are geographically dispersed from each other and from the health services that they need to access. We suspect, however, that in the long run, these extra resources will turn out to be highly cost-effective, and we hope that dimensions will be designed to assess the cost-effectiveness and cost benefit of this approach.

In any given population, it is reasonable to expect that a certain proportion will be responsive to interventions like ours, thus engaging in the behavior being promoted, and others will not. When the population is constrained, however, the results may be more circumspect; statistically speaking, when we constrain the variance, the associations among constructs will also be attenuated. In our study, the included population has been deliberately chosen to include only those most at risk for home-based delivery. A good proportion of this group is likely to be subjected to other constraints as well (we expect they will have lower levels of access to medical care, live further away from delivery sites, and have lower overall resources). Given this reality, we expect our overall effects to be lower in magnitude, as compared to other studies that have not constrained their sample to only the most at-risk groups. However, we believe this is an important population to reach, with tailored interventions that address their specific needs, because this group’s unique needs have mostly gone unaddressed (which is why they remain at-risk).

The HCD solutions we are implementing in this project (e.g., delivering audio devices and supporting print materials) are, indeed, rather labor intensive and involve the adoption and distribution of new hardware. This intensity and the tailored approach, we anticipate, will be impactful among women who are exposed to the intervention. From a scale-up perspective, however, this may appear to be an approach that the government would be reluctant to adopt, given the required training and resources. We note that this approach is not meant to be scaled up for everyone; rather, the idea is that, for women whose life circumstances put them at the highest levels of risk, we need alternative approaches: ones that may be cost intensive but are, nevertheless, cost-effective. We hope to shed light on this tension between intervention intensity and its scale-up potential through the findings and recommendations that will emerge from this study.

## Conclusions

Our study employs a sequential design to develop and evaluate an HCD-based intervention package aimed at improving maternal health service utilization among underserved pregnant women in rural Ethiopia. In Phase 1, we used a 5-stage HCD process to co-design solutions with women and stakeholders, fostering scalability and ownership within the health system in Phase 2. Our findings will provide important implications. First, it challenges the one-size-fits-all approach by focusing on the unique vulnerabilities of pregnant women, particularly those facing extreme poverty, food insecurity, and social isolation. This study advocates for initiatives that empower underserved populations to bridge maternal health gaps in the country. Second, we emphasized the role of husbands throughout our solutions, highlighting the need for gender-sensitive, women-centered programs to empower mothers, particularly in contexts where males are primary decision makers. Lastly, the study engaged community health workers, service providers, and government stakeholders in designing and implementing the intervention. Our findings may shed light on systems strengthening policies, including funding and technical support for rural health workers working in understaffed and inadequately resourced health facilities.

## Study status

Our study is currently at the data analysis stage of Phase 1, preparing for the phase 2 HCD process. We completed all HCD activities and assessments in Phase 1 by August 2024.

## Data Availability

No data are associated with this article. Data instruments and tools used in this project are available at OSF. Findings from this study will be disseminated through peer-reviewed scholarly publications. Data will be made available from the corresponding authors upon reasonable request. OSF: Enhancing maternal health service utilization among highly vulnerable pregnant women through a human-centered design process: Study protocol for a quasi-experimental study in Oromia, Ethiopia. https://doi.org/10.17605/osf.io/cmnps ^34^. This project contains the following underlying data: Appendix 1_Screening Tool. Pregnancy and vulnerability screening tool. Appendix 2_Survey questionnaire. Baseline and midline survey guide. Appendix 3_Qualitative guide Phase 1_Pregnant women. Qualitative questionnaire for pregnant women. Appendix 4–6_Qualitative guide Phase 1_Providers. Qualitative questionnaire for health care providers. OSF: SPIRIT Checklist for “Enhancing maternal health service utilization among highly vulnerable pregnant women through a human-centered design process: Study protocol for a quasi-experimental study in Oromia, Ethiopia.” https://doi.org/10.17605/osf.io/cmnps ^34^. Data are available under the terms of the Creative Commons Zero "No rights reserved" data waiver (CC0 1.0 Public domain dedication).
