# Supplementary material for: Developing and evaluating human-centered design solutions for enhancing maternal health service utilization among vulnerable pregnant women in Oromia, Ethiopia: Study protocol for a quasi-experimental study
Source: Gates Open Res. 2025 Feb 21;8:93. Originally published 2024 Aug 27. [Version 3] doi: 10.12688/gatesopenres.16277.3 (PMC11723881; doi:10.12688/gatesopenres.16277.3)
Supplement: Peer review history

# Reviewer report: Reviewer response for version 3

**DOI:** 10.21956/gatesopenres.17708.r39457

**Published:** 2025-02-26

**Authors:** Brooke A Levandowski

**Affiliations:** 1 Obstetrics & Gynecology, Medical Center, University of Rochester, Rochester, New York, USA

This version is much improved based on the reviewer comments and author responses. This is a complex, multi-level study, which has taken many crucial considerations into account. I look forward to learning results.

Is the study design appropriate for the research question?

Yes

Is the rationale for, and objectives of, the study clearly described?

Partly

Are sufficient details of the methods provided to allow replication by others?

No

Are the datasets clearly presented in a useable and accessible format?

Not applicable

Reviewer Expertise:

program evaluation; community based participatory research; maternal morbidity and mortality due to unsafe abortion in Kenya, Ethiopia, & Malawi

I confirm that I have read this submission and believe that I have an appropriate level of expertise to confirm that it is of an acceptable scientific standard.

# Reviewer report: Reviewer response for version 2

**DOI:** 10.21956/gatesopenres.17700.r39155

**Published:** 2025-01-10

**Authors:** Brooke A Levandowski

**Affiliations:** 1 Obstetrics & Gynecology, Medical Center, University of Rochester, Rochester, New York, USA

I appreciate all the work that has been completed in the revisions to respond to reviewer comments. 

 Please find additional comments below:

 1. Please update the Abstract so the Background and Methods sections are different and include the appropriate information.

 2. It would be helpful for an editor to review the language for correct English language. for example, the definition of "institutional delivery" has the wrong tense- occurred needs to change to occurs, or the whole sentence needs to be in past tense.

 3. Under study setting, since control and intervention arms were not selected at random, please share what characteristics were used to choose them.

 4. 2nd para under "development of HCD solutions": what is a self-aced program?

Is the study design appropriate for the research question?

Yes

Is the rationale for, and objectives of, the study clearly described?

Partly

Are sufficient details of the methods provided to allow replication by others?

No

Are the datasets clearly presented in a useable and accessible format?

Not applicable

Reviewer Expertise:

program evaluation; community based participatory research; maternal morbidity and mortality due to unsafe abortion in Kenya, Ethiopia, & Malawi

I confirm that I have read this submission and believe that I have an appropriate level of expertise to confirm that it is of an acceptable scientific standard, however I have significant reservations, as outlined above.

# Response

**Published:** 2025-02-20

**Authors:** Daryl Stephens

**Affiliations:** Health, Behavior and Society, Johns Hopkins University Bloomberg School of Public Health, Baltimore, Maryland, USA

Thank you, in response to your comments we have made the following amendments to the text:

1. We have corrected the duplicative abstract.
2. We have proofread the entire text for grammatical errors.
3. Study settings (woredas) were selected as intervention and control groups based on the following main characteristics: Agricultural livelihood, distance from major town, and maternal health service utilization. We have added this information to the Study Setting section. 
4. We have corrected this error in the sentence (to self-paced).

# Reviewer report: Reviewer response for version 2

**DOI:** 10.21956/gatesopenres.17700.r39243

**Published:** 2025-01-02

**Authors:** Syeda Tabeena Ali

**Affiliations:** 1 Public Health, Shaheed Zulfikar Ali Bhutto Institute of Science and Technology University, Karachi, Sindh, Pakistan

The study's rationale and objectives are clearly described, providing a comprehensive background on maternal health challenges in Ethiopia and justifying the use of human-centred design (HCD) to address them. The quasi-experimental design is appropriate, given the study's context and constraints, effectively balancing feasibility and methodological rigor.

 However, some methodological details are insufficient for full replication, particularly regarding the validation of the screening tool, justification for the cutoff score, and participant selection criteria for midline and end-line qualitative interviews.

 While the study does not present datasets, it would benefit from outlining a clear data-sharing plan to enhance transparency. Addressing these gaps, particularly through validation of the screening tool and elaborating on sampling and iterative processes, would strengthen the study's replicability and scientific soundness.

Is the study design appropriate for the research question?

Yes

Is the rationale for, and objectives of, the study clearly described?

Yes

Are sufficient details of the methods provided to allow replication by others?

Partly

Are the datasets clearly presented in a useable and accessible format?

Not applicable

Reviewer Expertise:

Public Health (Epidemiology and Biostatistics, Maternal and Child Health, Reproductive Health, Healthcare Violence/Gender-Based Violence, and Infectious Diseases)

I confirm that I have read this submission and believe that I have an appropriate level of expertise to confirm that it is of an acceptable scientific standard, however I have significant reservations, as outlined above.

# Response

**Published:** 2025-02-20

**Authors:** Daryl Stephens

**Affiliations:** Health, Behavior and Society, Johns Hopkins University Bloomberg School of Public Health, Baltimore, Maryland, USA

Thank you for your review. In response to your comments, we have made the following adjustments:

1. We have created a section titled “Maternal Vulnerability Segmentation Tool” under the Methods to outline the process of developing and validating the tool.
2. We have revised the “Recruitment” section to describe the participant selection criteria for qualitative interviews in both study phases.
3. We added information about data availability in the future dissemination of study findings.  

# Reviewer report: Reviewer response for version 2

**DOI:** 10.21956/gatesopenres.17700.r39154

**Published:** 2025-01-02

**Authors:** Kahabi Isangula

**Affiliations:** 1 School of Nursing and Midwifery, The Aga Khan University, Dar Es Salaam, Tanzania; 2 Muhimbili Research Centre, National Institute for Medical Reasearch, Dar Es Salaam, Tanzania

The authors have made significant improvements to the manuscript. However, I have noted that the information in the 'Background' and 'Methods' subsections of the Abstract is duplicative. I recommend revising these sections and cross-checking the document to ensure similar issues do not appear elsewhere. For reference:

 Abstract

 Background

*Disproportionate uptake of and access to maternal and child health services remain significant challenges across and within countries. Differing geographic, economic, environmental, and social factors contribute to varying degrees of vulnerabilities among individuals, which manifest as disparities in maternal and newborn health outcomes. Designing solutions according to need is vital to improve maternal and child health outcomes. In this paper, we describe our study protocol on developing and evaluating the effectiveness of human-centered design (HCD) solutions to improve maternal health service uptake among vulnerable pregnant women in rural areas of Ethiopia.*

 Methods

*Disproportionate uptake of and access to maternal and child health services remain significant challenges across and within countries. Differing geographic, economic, environmental, and social factors contribute to varying degrees of vulnerabilities among individuals, which manifest as disparities in maternal and newborn health outcomes. Designing solutions according to need is vital to improve maternal and child health outcomes. In this paper, we describe our study protocol on developing and evaluating the effectiveness of human-centered design (HCD) solutions to improve maternal health service uptake among vulnerable pregnant women in rural areas of Ethiopia.*

Is the study design appropriate for the research question?

Partly

Is the rationale for, and objectives of, the study clearly described?

Partly

Are sufficient details of the methods provided to allow replication by others?

Partly

Are the datasets clearly presented in a useable and accessible format?

Not applicable

Reviewer Expertise:

Maternal and child health, health service research, and human centred design

I confirm that I have read this submission and believe that I have an appropriate level of expertise to confirm that it is of an acceptable scientific standard, however I have significant reservations, as outlined above.

# Response

**Published:** 2025-02-20

**Authors:** Daryl Stephens

**Affiliations:** Health, Behavior and Society, Johns Hopkins University Bloomberg School of Public Health, Baltimore, Maryland, USA

Thank you, we have corrected the duplicative abstract.

# Reviewer report: Reviewer response for version 1

**DOI:** 10.21956/gatesopenres.17642.r38028

**Published:** 2024-10-11

**Authors:** Brooke A Levandowski

**Affiliations:** 1 Obstetrics & Gynecology, Medical Center, University of Rochester, Rochester, New York, USA

This is an interesting study that shares a unique method of addressing maternal and infant mortality, by asking women themselves within HCD constructs. I look forward to sharing the vulnerability scale and learning results.

 There are two points about the Introduction that may increase understanding of the Ethiopia setting.

 1. for the HSTP-II, what does it mean to "transform woredas toward zero home delivery"? (What is the level now?) or to "improve health system responsiveness"? (responsiveness to what?)

 2. The authors helpfully identified barriers to ANC care and hospital delivery at the individual, social, cultural, structural, and service-related levels. (suggest adding mention of delivery cost; only transportation costs noted). The intervention only appears to address a few individual level barriers while hypothesizing this will lead to systemic changes. I suggest aligning the hypothesis with individual level changes, as that may be more appropriate.

 There are a few points that I believe, if added, make this study more easily replicable.

 1. For those unfamiliar with Ethiopian geography, perhaps a figure or table could be used to show the relationship between regions, woredas, EAs, and PHCUs. It's unclear what the terms "below the zone" and "below the region" mean in the study setting description.

 2. How many women were recruited by woredas? How were the women chosen purposively for the midline interviews? Did/will interviewers go to the women's homes?

 3. What happened in the intervention if the husband wasn't home when the HDA arrived to deliver the intervention?

 4. How were women, husbands, &health workers compensated for HDA development, focus groups, and intervention home visits? 

 5. Why wasn't the pretest included in Phase 2?

Is the study design appropriate for the research question?

Yes

Is the rationale for, and objectives of, the study clearly described?

Partly

Are sufficient details of the methods provided to allow replication by others?

No

Are the datasets clearly presented in a useable and accessible format?

Not applicable

Reviewer Expertise:

program evaluation; community based participatory research; maternal morbidity and mortality due to unsafe abortion in Kenya, Ethiopia, & Malawi

I confirm that I have read this submission and believe that I have an appropriate level of expertise to confirm that it is of an acceptable scientific standard, however I have significant reservations, as outlined above.

# Response

**Published:** 2024-12-09

**Authors:** Daryl Stephens

**Affiliations:** Health, Behavior and Society, Johns Hopkins University Bloomberg School of Public Health, Baltimore, Maryland, USA

We are grateful to the reviewers for their thorough review of this manuscript. We provide descriptions about how we have modified the paper following point-by-point response below.

**1. For the HSTP-II, what does it mean to "transform woredas toward zero home delivery"? (What is the level now?) or to "improve health system responsiveness"? (responsiveness to what?)**

- The government’s push for zero home delivery is the overarching motto set by the Ministry of Health to mitigate home delivery at the community level. We have modified the phrase to avoid confusion. There is no publicly available data on institutional/home delivery at the woreda level, though we expect it to greatly vary across woredas. To highlight the disparity issue, we provided available statistics on regional disparities in the following paragraph.
- The HSTP-II defined health system responsiveness as “the level to which health services respond to the needs and expectations of targeted individuals and communities.” We have added the language to enhance clarity.

**2. The authors helpfully identified barriers to ANC care and hospital delivery at the individual, social, cultural, structural, and service-related levels. (suggest adding mention of delivery cost; only transportation costs noted). The intervention only appears to address a few individual level barriers while hypothesizing this will lead to systemic changes. I suggest aligning the hypothesis with individual level changes, as that may be more appropriate.**

- We appreciate this helpful suggestion. We have added delivery cost as one of the structural factors.
- We outlined a list of potential factors to emphasize the broad range of participant experiences we might gather through the HCD process, rather than to propose a hypothesis for change based on our solutions. This perspective was especially pertinent to the unique circumstances faced by our target population. Given the strength of HCD in enabling designers to remain open to emerging ideas from participants while avoiding predefined hypotheses, we were able to capture diverse experiences and challenges related to service uptake. However, while the insights generated spanned multiple levels, we had to exclude some structural-level solutions due to resource constraints. These details are described in the “Development of HCD Solutions” section. We also added a sentence on our expected level of change to enhance clarity.

**3.** **For those unfamiliar with Ethiopian geography, perhaps a figure or table could be used to show the relationship between regions, woredas, EAs, and PHCUs.**

 To enhance the reader’s understanding of the Ethiopian administration and health system structure, we created Figure 1. 

**4. How many women were recruited by woredas? How were the women chosen purposively for the midline interviews? Did/will interviewers go to the women's homes?**

 Pregnant women were not recruited by woreda. Rather, the recruitment was done per enumeration areas as indicated in the “Recruitment” section.

**5. What happened in the intervention if the husband wasn't home when the HDA arrived to deliver the intervention?**

 The HDA first tried to reschedule the home visit, but if the husband was unavailable for an extended period, the HDA facilitated discussions with other household members.

**6. How were women, husbands, & health workers compensated for HDA development, focus groups, and intervention home visits?**

 Compensation for participants varied by type of study participant and level of participation. For pregnant women engaged in focus group discussions (FGDs), refreshments were provided during the discussions, and we covered their transportation expenses for traveling from home to the meeting hall. For pregnant women participating in survey interviews, we provided two bars of soap. However, no compensation was offered to healthcare providers, as the interviews were conducted at their offices during their regular working hours.

**7. Why wasn't the pretest included in Phase 2?**

 The Phase 2 HCD solutions will be tested with two target populations (pregnant women and implementers).

# Reviewer report: Reviewer response for version 1

**DOI:** 10.21956/gatesopenres.17642.r37886

**Published:** 2024-09-06

**Authors:** Kahabi Isangula

**Affiliations:** 1 School of Nursing and Midwifery, The Aga Khan University, Dar Es Salaam, Tanzania; 2 Muhimbili Research Centre, National Institute for Medical Reasearch, Dar Es Salaam, Tanzania

Thank you for the opportunity to review the article titled *"Enhancing Maternal Health Service Utilization Among Highly Vulnerable Pregnant Women Through a Human-Centered Design Process: Study Protocol for a Quasi-Experimental Study in Oromia, Ethiopia,"* submitted to this journal. The topic is highly relevant, and the innovative use of human-centered design (HCD) is expected to produce solutions that are both practical and widely accepted by pregnant women. I have a few suggestions to improve the manuscript:

 1. **Title:**

 The title appears misleading and may need revision to clarify that the quasi-experimental design pertains specifically to one aspect of the HCD process—prototype validation—and not the entire HCD methodology. The early phases of HCD, such as co-design, may not involve a quasi-experimental design.

 If the protocol covers the entire HCD process, I suggest a title such as:

*"Co-Designing and Evaluating  Solutions for Enhancing Maternal Health Service Utilization Among Highly Vulnerable Pregnant Women: Study Protocol for Human-Centered Design in Oromia, Ethiopia."*

 If the protocol only covers the solution implementation phase, I suggest a title such as: 

*"Evaluating Co-Designed Solutions for Enhancing Maternal Health Service Utilization Among Highly Vulnerable Pregnant Women: Study Protocol for a Quasi-Experimental Study in Oromia, Ethiopia."*

*2.* **Abstract:**

**Background:** The objective is somewhat unclear. Is the aim to co-design and evaluate solutions, or to evaluate solutions that have already been co-designed? The latter implies that the solutions have been developed prior to the proposed study. If the latter is true, please clarify.

**Methods: **Significant restructuring is needed. The authors should briefly explain how the solutions will be developed using HCD before they are evaluated. Summarize the HCD steps, participants, and the process leading up to the validation study. It should also be clarified whether the solutions will be tested within the same population in which they were developed.

**Conclusions: **While a sequential approach is mentioned, it is not adequately described in the methods summary. Additionally, include a sentence about the expected prototypes and how the solutions are anticipated to impact practice and policy.

 3. Introduction 

 Given that HCD is used to address complex problems: (1) Highlight the complex interplay of factors influencing antenatal care (ANC) uptake among pregnant women; (2) Examine existing interventions aimed at addressing these factors, identifying the gaps that HCD can address; and (3) Explain why solutions designed through HCD are expected to drive meaningful changes. If the solutions already exist, explain what are they and how they were developed 

 4. Objectives:

 Please refer to the comments on the abstract regarding the objective. The HCD component—its iterative steps, objectives of each step, participants, and how each step builds on the previous one—is not clearly reflected in the objectives.  The authors should also clarify whether they plan to refine the solution based on findings from the quasi-experimental study. It may be helpful to refer to previous studies, such as Isangula K et a. (2022 [Ref - 1]) https://pubmed.ncbi.nlm.nih.gov/35797107/  OR  Isangula K et al. (2023 [Ref-2] https://bmcnurs.biomedcentral.com/articles/10.1186/s12912-023-01472-w.

 5. Methods

 Although HCD seems to be the backbone of this study, the description of how the solutions will be (or were) co-designed through HCD is insufficient. The focus appears to be more on the implementation/validation phase rather than the full HCD process. I recommend providing a detailed description of the HCD component used for solution development—its iterative steps, the objectives of each step, the participants involved, and how each step builds on the previous one. My understanding is that a community-based quasi-experimental design is being employed. If the solutions have already been developed, as suggested in the description of phase 1, the authors need to elaborate on how these solutions were reached through the HCD process during phase 1

 The paragraphs regarding the baseline survey, phase 1, and subsequent paragraphs are quite confusing. While HCD is mentioned, it’s unclear how it was implemented. I recommend providing a clearer explanation of the HCD process in phase 1, detailing how it was carried out, the participants of each step and their recruitments, the specific steps taken, and how it informed the development of the solutions.

 In summary, while the description of the experimental study itself is okay, a significant restructuring of the methods section is necessary to ensure clarity, coherence, and proper alignment with fundamental HCD principles.

**6. Discussion**

 The authors should elaborate on the expected results and explain how these will be compared to findings from previous studies.

**7. Conclusion**

 Please add a conclusion section. Expand on the content from the abstract, emphasizing how the findings are expected to inform both practice and policy.

 I believe these suggestions will result into improved and clear protocol.

Is the study design appropriate for the research question?

Partly

Is the rationale for, and objectives of, the study clearly described?

Partly

Are sufficient details of the methods provided to allow replication by others?

Partly

Are the datasets clearly presented in a useable and accessible format?

Not applicable

Reviewer Expertise:

Maternal and child health, health service research, and human centred design

I confirm that I have read this submission and believe that I have an appropriate level of expertise to confirm that it is of an acceptable scientific standard, however I have significant reservations, as outlined above.

## References

[1] : Strengthening Interpersonal Relationships in Maternal and Child Health Care in Rural Tanzania: Protocol for a Human-Centered Design Intervention. *JMIR Res Protoc* .2022;11(7) : 10.2196/37947 e37947 10.2196/37947 35797107 PMC9305451

[2] : Interventions co-designed by healthcare providers and clients for improving therapeutic relationships in maternal and child healthcare: a pilot study using human centered design in rural Tanzania. *BMC Nurs* .2023;22(1) : 10.1186/s12912-023-01472-w 314 10.1186/s12912-023-01472-w 37704982 PMC10500767

# Response

**Published:** 2024-12-09

**Authors:** Daryl Stephens

**Affiliations:** Health, Behavior and Society, Johns Hopkins University Bloomberg School of Public Health, Baltimore, Maryland, USA

We are grateful to the reviewers for their thorough review of this manuscript. We provide descriptions about how we have modified the paper following point-by-point response below.

**1. Title**

 We appreciate the author’s helpful suggestion. We have revised the title of our paper as follows: *“Developing and evaluating human-centered design solutions for enhancing maternal health service utilization among vulnerable pregnant women in Oromia, Ethiopia: Study protocol for a quasi-experimental study”*

**2. Abstract**

**Background:**

 Solutions were already developed for Phase 1, and solutions for Phase 2 are under development. As our protocol covers both the development and evaluation processes, we have revised our study objective in the Background section of Abstract accordingly.

**Methods:**

 We appreciate the reviewer’s suggestion. We have reconfigured the Methods section in the Abstract by study phase to enhance the reader’s understanding of our two-phased design. Given the word limit in the Abstract, we have provided detailed HCD procedures in the main text. 

**Conclusions:**

 We have clarified the sequential approach along with our finalized solutions in the Methods section. We have also added a sentence about our expected implications for practice and policy in the Conclusions and Implications section.

**3. Introduction**

 We have added a paragraph to the Introduction section, detailing HCD’s potential in addressing multilevel factors of health issues and its application in prior interventions. We also described why HCD would be particularly useful for our study.

**4. Objectives**

 To enhance the understanding of our study objectives, we divided our objectives into intervention development and intervention evaluation. Our study objectives remain at the overall study design level, rather than within the HCD process, which we described in the Methods section more in detail.

**5. Methods**

- A detailed description of the HCD process, including participants recruitment, testing, and solution finalization were added on the “HCD workshop procedure” and “Development of HCD solutions” sections. Throughout these sections, HCD goals and methods for in Phases 1 and 2 have been clarified.
- In terms of structure, the overall Methods section is organized to follow a quasi-experimental trial designed to evaluate HCD solutions. The trial description includes information about both intervention and control arms, pertaining to sample, study design, data collection, and setting for the trial.
- All HCD-related information is provided under the “Intervention” section. Since our HCD activities only occurred for the intervention arm, we believe this structure aligns with the overall flow of this paper.

**6. Discussion**

 We added a paragraph in the Discussion section to address this important issue. See paragraph, “In any given population,….”

**7. Conclusion**

 We appreciate the author’s suggestion to highlight the practice and policy implications of our study. We added a conclusion section at the end of our manuscript.
